# Supplementary material for: No evidence for differential gene expression in major depressive disorder PBMCs, but robust evidence of elevated biological ageing
Source: Transl Psychiatry. 2021 Jul 22;11:404. doi: 10.1038/s41398-021-01506-4 (PMC8298604; doi:10.1038/s41398-021-01506-4)
Supplement: Supplementary file 1 — Supplementary Information [file 41398_2021_1506_MOESM1_ESM.docx]

**Supplementary Information**

**Data on RNS sequencing are deposited in the University of Cambridge:**

[**https://www.repository.cam.ac.uk/handle/1810/323434**](https://www.repository.cam.ac.uk/handle/1810/323434)

**Supplementary Table 1.** Selection of clinical characteristics for all HC and MDD samples.

**Supplementary Table 2.** Enriched (FDR < 5%) gene ontologies for the 50 most significant genes (mean p value) amongst the 50 comparisons of randomised cases and controls.

**Methods**

***Participants.*** This was a non-interventional study, conducted as part of the Wellcome Trust Consortium for Neuroimmunology of Mood Disorders and Alzheimer’s disease (NIMA). There were five clinical study centres in the UK: Brighton, Cambridge, Glasgow, King’s College London, and Oxford. All procedures were approved by an independent Research Ethics Committee (National Research Ethics Service East of England, Cambridge Central, UK) and the study was conducted according to the Declaration of Helsinki. All participants provided informed consent in writing and received £100 compensation for taking part.

**Sample and Eligibility Criteria.** For all participants, the following inclusion criteria applied: age 25-50 years, able to give informed consent; able to fast for 8 hours, and abstain from strenuous exercise for 72h, prior to venous blood sampling; and fluent English. The following exclusion criteria applied: pregnancy or breast feeding; alcohol or substance use disorder in the preceding 12 months; participation in an investigational drug study within the preceding 12 months; lifetime history of any medical disorder or current use of any medication (e.g. statins, corticosteroids, antihistamines, anti-inflammatory medications).

Adult patients meeting Diagnostic and Statistical Manual Version 5 (DSM-5) criteria for MDD (American Psychiatric Association, 2012) were recruited from NHS mental health and primary care services and from the general population by purposive advertising. Lifetime histories of bipolar disorder or non-affective psychosis were additional exclusion criteria. Diagnosis of MDD and other psychiatric disorders was ascertained by Structured Clinical Interview for DSM-5^1^. Current depressive symptom severity was defined using total scores from the 17-item Hamilton Rating Scale for Depression (HAM-D)^2^, and lifetime anti-depressant medication use was quantified using the Antidepressant Treatment Response Questionnaire (ATRQ)^3^. Cut-offs were defined *a priori* based on the literature. Total HAM-D score > 17 is a standard threshold for entry into placebo-controlled treatment trials of MDD; whereas a lower threshold of total HAM-D > 13 is typically used to define treatment-resistant depression, because there is usually some modest symptomatic response to treatment even if patients remain depressed^4, 5^. A group of healthy volunteers was recruited by advertising with no current or past history of any major psychiatric disorder as defined by DSM-5, and no history of monoaminergic drug treatment for any indication. Healthy volunteers completed the same screening and baseline assessments as patient groups (see below). Age, gender, medical history, smoking status, and family history were documented by semi-structured clinical interviews. Height and weight were measured for calculation of BMI (kg/m2).

***Questionnaire assessments.*** Psychological symptoms and childhood adversity were assessed as previously described^6^ by administration of the following questionnaires: the Beck depression inventory (BDI v2.0)^7^; the Spielberger State-Trait Anxiety Rating scale (STAI)^8^; the Chalder Fatigue Score (CFS)^9^; the Snaith-Hamilton Pleasure Scale (SHAPS)^10^; and the Childhood Trauma Questionnaire (CTQ)^11^.

***Sampling and isolation of PBMCs***

Whole blood was collected in K2EDTA tubes (BD, USA) by peripheral venepuncture and allowed to cool to room temperature for a minimum of 45 minutes. Blood was diluted in DPBS (Life Technologies, USA) before being added to Leucosep tubes (Greiner Bio-One Ltd, Austria) containing Histopaque (Sigma-Aldrich, USA) for density gradient centrifugation. Leucosep tubes were centrifuged at 800g for 15 minutes at room temperature with the break off. The interphase layer containing peripheral blood mononuclear cells (PBMCs) was carefully retrieved and washed twice in DPBS. Cells were counted and 1x10^6^ PBMCs were resuspended in 350ul RLTplus buffer (Qiagen, Germany) containing 10ul/ml 2-Mercaptoethanol (Sigma-Aldrich, USA). Cell suspension was loaded on to a QIAshredder (Qiagen, Germany) and centrifuged at >10,000g for 2 minutes for homogenisation. Lysed cell suspension was stored at -80°C prior to RNA extraction. RNA was extracted using the RNeasyMini Kit (Qiagen, Germany) as per the manufacturer’s instructions. In brief, lysed cell samples were thawed on ice and diluted 1:1 with 70% ethanol to precipitate the RNA, before being transferred to an RNeasy spin column and subjected to a series of washes and spins. RNA was eluted in 50ul RNase-free H_2_O and stored at -80°C before being sent for sequencing.

***RNA-sequencing and processing.*** PBMC samples were taken from four separate population groups. Numbering **44** healthy controls, **94** MDD treatment-resistant, **47** MDD treatment-responsive, **46** MDD untreated patients. The distribution is illustrated in **figure 1a**. All PBMC samples had an RNA Integrity Number (RIN) ≥ 8 and were analysed for gene expression levels by RNA-Seq. Sequencing was performed by Edinburgh Genomics. Libraries were generated using a TruSeq stranded mRNA-seq library preparation kit and was sequenced on a HiSeq 4000 using 75bp paired end reads. Samples were sequenced to an average depth of 54.5 million read pairs. Reads were trimmed using Cutadapt 1 (version cutadapt-1.9.dev2)^12^. Reads were trimmed for quality at the 3’ end using a quality threshold of 30 and for adapter sequences of the TruSeq stranded mRNA-seq kit (AGATCGGAAGAGC). Reads after trimming were required to have a minimum length of 50. The reference used for mapping was the Homo sapiens genome from Ensembl, assembly GRCh38, annotation version 84. Reads were aligned to the reference genome using STAR 2 (version 2.5.2b)^13^ specifying paired-end reads and the option --outSAMtype BAM Unsorted. All other parameters were left at default. Reads were assigned to features of type ‘exon’ in the input annotation grouped by gene_id in the reference genome using featureCounts 3 (version 1.5.1). Strandedness was set to ‘reverse’ and a minimum alignment quality of 10 was specified. After filtering for only protein coding genes, we observed a median of **40** million exonic aligned reads per sample (>85%).

***RNA-sequencing differential expression analysis.*** Differential expression analysis was performed using DESeq2 (version 1.18.1)^14^. The count matrix was initially filtered to include only coding genes with a mean of > 1 read per sample. For the comparisons of binary clinical covariates (e.g. gender, tobacco) one group was compared to the other. For continuous clinical covariates (e.g. age, BMI) the patients in the lower quartile were compared to those in the upper quartile. No additional covariates were used in the DEseq2 model when comparing clinical covariates. For the differential comparisons between HC and the MDD groups the 15 confounding clinical covariates (**Figure 1b**) (identified as having > 5 significant associated genes (adjusted p < 0.01)) and “batch” were included as covariates in the model. To control for extreme outlier values typical in large and heterogeneous datasets, a Cooks cut-off of 0.2 was used. All other parameters were left to default. Significance was set at an adjusted p of < 0.01 in all cases.

***Correction of RNA-sequencing expression data.*** The base expression values were generated with DEseq2, using the “counts” function with “normalized” set to “true”. These values were used in the Cibersort analysis. When plotting clinical covariates (e.g. age, gender and BMI), and when performing the biological age meta-gene analysis, extreme outliers were controlled for by capping individual expression values at 1.5 times the interquartile range, on a gene by gene basis. When plotting the expression values for the HC and MDD groups and for the co-expression analysis the expression data was additionally corrected using Combat^15^, to control the effects of the 15 confounding clinical confounders (**Figure 1b**) and “batch” (prior to capping at 1.5 times the interquartile range). All other parameters were left to default.

***RNA-sequencing randomised cases and controls.*** To generate the differential signatures for randomised cases and controls, the 231 PBMC samples were randomised using the r function “sample” (without replacement). The number of ‘cases’ and ‘controls’ in the randomised groups was identical to the real groups (**44** HC and **187** MDD). Differential expression was performed as the real HC vs MDD comparison (including using the 15 clinical covariates and “batch” in the linear model) using DESeq2, as described above. The randomising of groups followed by differential expression analysis was repeated fifty times, using random seeds 1-50 for reproducibility. To generate the p value distribution for the waterfall plot each of the fifty comparisons were sorted by descending p value, and then averaged at each rank. The 250 lowest p-values were then plotted. To calculate the median and maximum number of false positive genes at a given adjusted p threshold the median and maximum across all 50 randomised comparisons was used. To generate the 50 most significant genes the 50 differential expression files (randomised comparisons) were merged and the per gene mean p-value calculated. The genes were then sorted by mean p-value and the 50 lowest used for further analysis. These 50 genes were then used in a DAVID over-representation (DAVID functional enrichment (version 6.8)^16, 17^) with the background of PBMC expressed coding genes and selecting GOTERM_BP_DIRECT **(Supplementary Table 2)**. All other parameters were left to default.

***Co-expression analysis.*** The co-expression network cluster analysis was based on the analysis performed by Le et al^18^ and used their code as a template. Firstly, we removed genes with low expression (mean > 10, in the Combat corrected data) or with exceptionally high coefficient of variability (standard deviation / mean < 0.15), to reduce the chance that correlations could be driven by technical variability. Next the resultant expression matrix was converted into a matrix of gene vs gene Pearson correlation coefficients (PCC) using the R function “cor”. This was then filtered to include only genes that correlate with at least one other gene at PCC > 0.5. This was then converted into a list of co-expression network edges. A topological overlap matrix (TOM) for the resultant network was generated using the R method “TOMsimilarity” with “TOMtype=unsigned”. A hierarchical tree was constructed from the inversed TOM matrix (i.e. dissimilarity), using “hclust” with average reordering. The tree was then cut at a height of 0.95 to generate clusters of correlating genes. In order to focus only on large and reliable clusters a minimum cluster size of 50 genes was set. This created **48** clusters. To identify any clusters with significantly different gene expression between HC and MDD samples, a metagene for each cluster was generated. To do so, the expression values for all genes were scaled into per gene Z-scores. Next, for each cluster the mean expression z-score across all genes in that cluster was calculated, for each sample. Finally, for each cluster the resultant scores for the HC samples were compared to that of the MDD samples using an unpaired, two-tailed T-test. The resultant p values were adjusted using the Benjamini-Hochberg procedure. No cluster was significant at adjusted p < 0.25.

***Expression microarray analysis.*** The GSK-HiTDiP^19^ MDD microarray data was downloaded from GEO (GSE98793) and the 22 samples that were reported to have failed QC were removed. The expression data was then quantile normalised using Limma^20^. Unannotated probe sets were removed. To control for genes represented by several different probe sets, Jetset^21^ was used to select the probe set for each gene with the highest Jetset score. This resulted in 20,191 valid probe sets. Differential expression analysis was performed between the HC and MDD groups using Limma, and included batch, age, gender and anxiety as additional covariates. All other parameters were left to default. The quantile normalised expression values were corrected for batch using Limmas “removeBatchEffect” function.

***RNA-sequencing biological age meta-genes.*** The analysis aimed to determine whether MDD samples had altered progression relative to HC along a shared age expression phenotype, and had three steps. (1) Identification of a list of genes associated with age in PBMCs, (2) calculation of the per sample age meta-gene expression values – i.e. an estimate of biological ageing, (3) statistical analysis comparing the meta-gene expression against chronological age for HC and MDD separately.

1. A list of PBMC age associated genes was identified by using Deseq2 to compare the samples of lowest to highest quartile of age, as described previously. Note – this differential model used both HC and MDD samples to identify PBMC age associated genes, in order to increase power. This assumed that the underlying mechanisms of ageing were unaltered between HC and MDD samples. An assumption supported by the absence of any differentially expressed genes between HC and MDD.
2. To estimate the correlation between biological and chronological age the expression values (non-corrected but outlier capped) for the PBMC age related genes were scaled (per gene z-score), with the sign inversed for genes that were downregulated with age. Next, the mean scaled value (across all significant genes) was calculated per sample. This meta-gene value was considered as the samples biological age. Biological age was then correlated (Spearman Correlation Coefficient (SCC)) against chronological age (across all samples) to give a correlation value. To identify an optimal set of age-related genes a range of p-value and fold change cut-offs was used when selecting significant genes. P-values ranged from 0.1 to 0.001 and absolute log^2^ fold change from 0.1 to 2. At each cut-off combination the biological age and SCC was calculated. The combination that resulted in the highest SCC (0.72) was used. The analysis was replicated using the GSK-HiTDIP whole blood dataset, where the optimal SCC was 0.49.
3. To determine the difference in biological and chronological age a linear model was used. The model was biological age ~ chronological age. The resultant residuals were used to establish differences in biological age by HC or MDD group.

***Heatmaps.*** Heatmaps were generated using the R package hclust (version 1.1.25) with distance_method = "spearman", clustering_method = "average" and reorder_function = "average". Row scaled (z-score) expression values were used.

1. First M, Spitzer R, Gibbon M, Williams J. Structured clinical interview for DSM-IV axis I disorders—clinician version (SCID-CV). Washington DC, and London, England: American Psychiatry Association Press. Inc1997.

2. Hamilton M. A rating scale for depression. *J Neurol Neurosurg Psychiatry* 1960; **23:** 56-62.

3. Desseilles M, Witte J, Chang TE, Iovieno N, Dording CM, Ashih H *et al.* Assessing the adequacy of past antidepressant trials: a clinician's guide to the antidepressant treatment response questionnaire. *J Clin Psychiatry* 2011; **72**(8)**:** 1152-1154.

4. Katona CL, Robertson MM, Abou-Saleh MT, Nairac BL, Edwards DR, Lock T *et al.* Placebo-controlled trial of lithium augmentation of fluoxetine and lofepramine. *Int Clin Psychopharmacol* 1993; **8**(4)**:** 323.

5. Marcus RN, McQuade RD, Carson WH, Hennicken D, Fava M, Simon JS *et al.* The efficacy and safety of aripiprazole as adjunctive therapy in major depressive disorder: a second multicenter, randomized, double-blind, placebo-controlled study. *J Clin Psychopharmacol* 2008; **28**(2)**:** 156-165.

6. Chamberlain SR, Cavanagh J, de Boer P, Mondelli V, Jones DNC, Drevets WC *et al.* Treatment-resistant depression and peripheral C-reactive protein. *Br J Psychiatry* 2019; **214**(1)**:** 11-19.

7. Beck AT, Steer RA, Brown GK. Manual for the beck depression inventory-II. *San Antonio, TX: Psychological Corporation* 1996; **1:** 82.

8. Spielberger CD. STAI manual for the state-trait anxiety inventory. *Self-Evaluation Questionnaire* 1970**:** 1-24.

9. Chalder T, Berelowitz G, Pawlikowska T, Watts L, Wessely S, Wright D *et al.* Development of a fatigue scale. *Journal of psychosomatic research* 1993; **37**(2)**:** 147-153.

10. Snaith R, Hamilton M, Morley S, Humayan A, Hargreaves D, Trigwell P. A scale for the assessment of hedonic tone the Snaith–Hamilton Pleasure Scale. *The British Journal of Psychiatry* 1995; **167**(1)**:** 99-103.

11. Bernstein DP, Fink L, Handelsman L, Foote J, Lovejoy M, Wenzel K *et al.* Initial reliability and validity of a new retrospective measure of child abuse and neglect. *The American journal of psychiatry* 1994.

12. Martin M. Cutadapt removes adapter sequences from high-throughput sequencing reads. *2011* 2011; **17**(1)**:** 3.

13. Dobin A, Davis CA, Schlesinger F, Drenkow J, Zaleski C, Jha S *et al.* STAR: ultrafast universal RNA-seq aligner. *Bioinformatics* 2012; **29**(1)**:** 15-21.

14. Love MI, Huber W, Anders S. Moderated estimation of fold change and dispersion for RNA-seq data with DESeq2. *Genome Biology* 2014; **15**(12)**:** 550.

15. Leek JT, Johnson WE, Parker HS, Fertig EJ, Jaffe AE, Storey JD *et al.* sva: Surrogate variable analysis. *R package version* 2017; **3**(0)**:** 10.18129.

16. Huang da W, Sherman BT, Lempicki RA. Systematic and integrative analysis of large gene lists using DAVID bioinformatics resources. *Nat Protoc* 2009; **4**(1)**:** 44-57.

17. Huang da W, Sherman BT, Lempicki RA. Bioinformatics enrichment tools: paths toward the comprehensive functional analysis of large gene lists. *Nucleic Acids Res* 2009; **37**(1)**:** 1-13.

18. Le TT, Savitz J, Suzuki H, Misaki M, Teague TK, White BC *et al.* Identification and replication of RNA-Seq gene network modules associated with depression severity. *Transl Psychiatry* 2018; **8**(1)**:** 180.

19. Leday GGR, Vertes PE, Richardson S, Greene JR, Regan T, Khan S *et al.* Replicable and Coupled Changes in Innate and Adaptive Immune Gene Expression in Two Case-Control Studies of Blood Microarrays in Major Depressive Disorder. *Biol Psychiatry* 2018; **83**(1)**:** 70-80.

20. Ritchie ME, Phipson B, Wu D, Hu Y, Law CW, Shi W *et al.* limma powers differential expression analyses for RNA-sequencing and microarray studies. *Nucleic Acids Research* 2015; **43**(7)**:** e47-e47.

21. Li Q, Birkbak NJ, Gyorffy B, Szallasi Z, Eklund AC. Jetset: selecting the optimal microarray probe set to represent a gene. *BMC Bioinformatics* 2011; **12**(1)**:** 474.
