# Supplementary material for: No evidence for differential gene expression in major depressive disorder PBMCs, but robust evidence of elevated biological ageing
Source: Transl Psychiatry. 2021 Jul 22;11:404. doi: 10.1038/s41398-021-01506-4 (PMC8298604; doi:10.1038/s41398-021-01506-4)
Supplement: Supplementary file 2 — Full author list supplemental [file 41398_2021_1506_MOESM2_ESM.docx]

**No evidence for differential gene expression in major depressive disorder PBMCs, but robust evidence of accelerated biological ageing.**

John J. Cole, Alison McColl, Robin Shaw, Mary-Ellen Lynall, Philip J. Cowen, Peter de Boer, Wayne C. Drevets, Neil Harrison, Carmine Pariante, Lind Pointon, NIMA consortium, Carl Goodyear, Edward Bullmore and Jonathan Cavanagh

(PI indicates principal investigator and EC indicates executive committee member):

**Cambridge**: Edward T. Bullmore (PI, EC)^1,2,11^, Junaid Bhatti^1^, Samuel R. Chamberlain^1,2^, Marta M. Correia^1,12^, Amber Dickinson*, Andy Foster^2^, Manfred Kitzbichler^1^, Clare Knight^2^, Mary-Ellen Lynall^1^, Christina Maurice^1^, Howard Mount^13^, Ciara O’Donnell^1^, Linda J. Pointon^1^, Peter St George Hyslop^1,13,14^, Lorinda Turner^1^, Barry Widmer^1^, Guy B. Williams^1,14^

**Cardiff:** B. Paul Morgan (PI)^15^, Claire Leckey^15^, Angharad Morgan^15^, Caroline O’Hagan*, Samuel Touchard^15^

**Glasgow:** Jonathan Cavanagh (PI, EC)^3^, John J. Cole^3^, Catherine Deith*, Carl Goodyear^3^, John McClean^16^, Alison McColl^3^, Andrew McPherson*, Paul Scouller*, Robin Shaw, Murray Sutherland^16^

**Independent advisor**: H.W.G.M. (Erik) Boddeke (EC)^17^

**GlaxoSmithKline:** Jill Richardson (EC)^18^, Shahid Khan^11^, Phil Murphy^19^, Christine Parker^19^, Jai Patel^11^

**Janssen:** Declan Jones (EC)^6^, Peter de Boer^4^ (deceased) John Kemp^4^, Paul Acton^6^, Wayne C. Drevets^6^, Jeffrey S. Nye (deceased), Gayle Wittenberg^6^, John Isaac^6^, Anindya Bhattacharya^6^, Nick Carruthers^6^, Hartmuth Kolb^6^

**King’s College London:** Carmine Pariante (PI)^10^, Gareth Barker^20^, Heidi Byrom^10^, Diana Cash^20^, Antony Gee^20^, Caitlin Hastings^10^, Nicole Mariani^10^, Anna McLaughlin^10^, Valeria Mondelli^10^, Maria Nettis^10^, Naghmeh Nikkheslat^10^, Karen Randall^20^, Hannah Sheridan*, Camilla Simmons^20^, Nisha Singh^20^, Federico Turkheimer^20^, Victoria Van Loo*, Marta Vicente Rodriguez^20^, Tobias Wood^20^, Courtney Worrell*, Zuzanna Zajkowska*

**Lundbeck**: Niels Plath (EC)^21^, Jan Egebjerg^21^, Hans Eriksson^21^, Francois Gastambide^21^, Karen Husted Adams^21^, Ross Jeggo^21^, Christian Thomsen^21^, Jason O’Connor^22^, Jan Torleif Pederson^21^, Brian Campbell*, Thomas Möller*, Bob Nelson*, Stevin Zorn*

**Oxford:** Mary Jane Attenburrow (PI)^7,23^, Alison Baird, Jithen Benjamin^23^, Stuart Clare^25^, Philip Cowen^7^, I-Shu (Dante) Huang^24^, Samuel Hurley*, Helen Jones^23^, Simon Lovestone^7^, Francesca Mada^23^, Alejo Nevado-Holgado^7^, Akintayo Oladejo*, Elena Ribe^7^, Anviti Vyas*

**Pfizer:** Zoe Hughes (EC)^26^, Rita Balice-Gordon*, Brendon Binneman^26^, James Duerr^26^, Terence Fullerton^26^, Justin Piro^26^, Tarek Samad^26^, Jonathan Sporn^26^

**Southampton:** Hugh Perry (PI)^27^, Madeleine Cleal*, Gemma Fryatt^27^, Diego Gomez-Nicola^27^, Renzo Mancuso^27^

**Sussex:** Neil Harrison (PI, EC)^28^, Mara Cercignani^28^, Charlotte Clarke^28^, Elizabeth Hoskins^29^, Charmaine Kohn^29^, Rosemary Murray*, Dominika Wlazly^30^

^1^Department of Psychiatry, School of Clinical Medicine, University of Cambridge, CB2 0SZ, UK

^2^Cambridgeshire and Peterborough NHS Foundation Trust, Cambridge, CB21 5EF, UK

^3^Institute of Infection, Immunity & Inflammation, University of Glasgow, Sir Graeme Davies Building, Glasgow, G12 8TA, UK

^4^Neuroscience, Janssen Research & Development, Janssen Pharmaceutica NV, Turnhoutseweg 30, B-2340, Beerse, Belgium

^5^The Maurice Wohl Clinical Neuroscience Institute, Cutcombe Road, London, SE5 9RT, UK

^6^Neuroscience, Janssen Research & Development, LLC, Titusville, New Jersey, 08560, USA

^7^University of Oxford Department of Psychiatry, Warneford Hospital, Oxford, OX3 7JX, UK

^8^Brighton & Sussex Medical School, University of Sussex, Brighton, BN1 9RR, UK

^9^Sussex Partnership NHS Foundation Trust, Swandean, BN13 3EP, UK

^10^Stress, Psychiatry and Immunology Laboratory & Perinatal Psychiatry, Maurice Wohl Clinical Neuroscience Institute, King’s College London, SE5 9RT, UK

^11^Immuno-Psychiatry, Immuno-Inflammation Therapeutic Area Unit, GlaxoSmithKline R&D, Stevenage SG1 2NY, UK

^12^MRC Cognition and Brain Sciences Unit, 15 Chaucer Road, Cambridge CB2 7EF, UK

^13^Tanz Centre for Research in Neurodegenerative Diseases, 60 Leonard Avenue, Toronto, Ontario M5T 2S8 Canada

^14^Department of Clinical Neurosciences, University of Cambridge, CB2 0SZ, UK

^15^University of Cardiff, Cardiff CF10 3AT, UK

^16^NHS Greater Glasgow and Clyde, 1055 Great Western Rd, Glasgow G12 0XH, UK

^17^University of Groningen, 9712 CP Groningen, Netherlands

^18^Neurosciences Virtual PoC DPU, GlaxoSmithKline R&D, Stevenage SG1 2NY, UK

^19^Experimental Medicine Imaging, GlaxoSmithKline R&D, Stevenage SG1 2NY, UK

^20^Centre for Neuroimaging Sciences, Denmark Hill, London SE5 9AF, UK

^21^H. Lundbeck A/S Ottiliavej 9, 2500, Valby, Denmark

^22^University of Texas Health Science Center at San Antonio, San Antonio, Texas 78229, USA

^23^NIHR Oxford Cognitive Health Clinical Research Facility, Warneford Hospital, Oxford, OX3 7JX, UK

^24^The Kennedy Institute of Rheumatology, Oxford OX3 7FY, UK

^25^Oxford Centre for Functional MRI of the Brain, John Radcliffe Hospital, Oxford OX3 9DU, UK

^26^Pfizer, Inc., Cambridge, Massachusetts, USA

^27^Centre for Biological Sciences, University of Southampton, Southampton, UK

^28^Clinical Imaging Sciences Centre, University of Sussex, Brighton, BN1 9RR, UK

^29^Sussex Partnership NHS Foundation Trust, Hove BN3 7HZ, UK

^30^Brighton & Sussex University Hospitals NHS Trust, Brighton BN2 5BE, UK

*Former consortium members.
