# Supplementary material for: No evidence for differential gene expression in major depressive disorder PBMCs, but robust evidence of elevated biological ageing
Source: Transl Psychiatry. 2021 Jul 22;11:404. doi: 10.1038/s41398-021-01506-4 (PMC8298604; doi:10.1038/s41398-021-01506-4)
Supplement: Supplementary file 3 — Supplementary Table 1 [file 41398_2021_1506_MOESM3_ESM.pdf]

Supplementary Table 1. Selection of samples and clinical characteristics.

	**Control** (n=44)	**MDD Resistant** (n=94)	**MDD Responder** (n=47)	**MDD Untreated** (n=46)	**P-Value**	**Total** (n=231)
**Age (Years)**						
Mean	36.6	37.9	37.8	36.9	0.74[A]	37.5
(SD)	(7.12)	(7.49)	(8.11)	(8.50)		(7.73)
**Sex**						
Male	11 (25.0%)	28 (29.8%)	15 (31.9%)	15 (32.6%)		69 (29.9%)
Female	33 (75.0%)	66 (70.2%)	32 (68.1%)	31 (67.4%)	0.9[B]	169 (70.1%)
**BMI (kg/m^2)**						
Mean	25.5	27.6	27.9	26.4	0.18[A]	27.0
(SD)	(4.97)	(7.33)	(5.49)	(4.73)		(6.13)
Missing	2 (4.5%)	2 (2.1%)	1 (2.1%)	0 (0%)		5 (2.2%)
**Hamilton Score**						
Mean	0.86	18.16	4.09	20.09	**<0.0001**[C]	12.38
(SD)	(1.23)	(3.71)	(2.75)	(2.69)		(8.58)
**Alcohol Use**	22 (50.0%)	62 (66.0%)	32 (68.1%)	32 (69.6%)	0.2[B]	148 (64.1%)
**Tobacco Use**	13 (29.6%)	36 (38.3%)	13 (27.7%)	15 (32.6%)	0.6[B]	77 (33.3%)
**Drug Use (any)**	23 (52.3%)	43 (45.7%)	27 (57.5%)	21 (45.7%)	0.5[B]	114 (49.4%)
**Life Events Questionnaire Z-score**						
Mean	-0.499	0.167	0.012	0.138	**0.0021**[A]	0.000
(SD)	(0.541)	(1.090)	(0.991)	(1.033)		(1.000)
Missing	0 (0%)	3 (3.2%)	0 (0%)	1 (2.2%)		4 (1.7%)
**Infections**	7 (15.9%)	30 (32.3%)	17 (36.2%)	15 (32.6%)	0.1[B]	69 (30.0%)
Missing	0 (0%)	1 (1.1%)	0 (0%)	0 (0%)		1 (0.4%)
**Chalder Fatigue Scale**						
Mean	11.57	21.09	14.61	19.65	**<0.0001**[C]	17.64
(SD)	(3.19)	(5.54)	(5.35)	(5.23)		(6.33)
Missing	0 (0%)	1 (1.1%)	1 (2.1%)	3 (6.5%)		5 (2.2%)
**Beck's Depression Inventory**						
Mean	1.48	24.74	9.19	21.36	**<0.0001**[C]	16.45
(SD)	(1.57)	(9.69)	(8.25)	(7.30)		(12.2)
Missing	2 (4.5%)	3 (3.2%)	0 (0.0%)	2 (4.3%)		7 (3.0%)
**Childhood Trauma Score**						
Mean	62.40	62.09	62.44	62.07	0.9924[C]	62.21
(SD)	(4.85)	(10.48)	(8.68)	(7.49)		(8.63)
Missing	1 (2.3%)	5 (5.3%)	4 (8.5%)	2 (4.3%)		12 (5.2%)
**State-Trait Anxiety Inventory**						
Mean	91.67	88.50	86.79	88.74	0.1181[C]	88.83
(SD)	(8.40)	(8.93)	(8.99)	(10.72)		(9.28)
Missing	2 (4.5%)	4 (4.3%)	5 (10.6%)	4 (8.7%)		15 (6.5%)
**Snaith-Hamilton Pleasure Scale**						
Mean	16.68	32.56	22.65	31.62	**<0.0001**[C]	27.27

(SD)	(3.86)	(7.30)	(6.86)	(5.68)		(9.02)
Missing	0 (0%)	3 (3.2%)	1 (2.1%)	1 (2.2%)		5 (2.2%)

P-Value in **bold** denotes significance after Bonferroni correction. Test type: A = one-way ANOVA; B = Pearson's Chi-squared Test; C = Poisson regression.
