# Supplementary material for: No evidence for differential gene expression in major depressive disorder PBMCs, but robust evidence of elevated biological ageing
Source: Transl Psychiatry. 2021 Jul 22;11:404. doi: 10.1038/s41398-021-01506-4 (PMC8298604; doi:10.1038/s41398-021-01506-4)
Supplement: Supplementary file 4 — Supplemenary Table 2 [file 41398_2021_1506_MOESM4_ESM.pdf]

Supplementary Table 2.

ID	Term	Term Size	Overlap	Enrichment	FDR (%)
GO:0009615	response to virus	110	6	19.91	0.01564
GO:0060337	type I interferon signaling pathway	64	5	28.52	0.03642
GO:0071347	cellular response to interleukin-1	71	5	25.71	0.05493
GO:0071356	cellular response to tumor necrosis factor	110	5	16.59	0.30213
GO:0060326	cell chemotaxis	65	4	22.46	0.99735
GO:0070098	chemokine-mediated signaling pathway	71	4	20.57	1.28872
GO:0051607	defense response to virus	165	5	11.06	1.39278
GO:0006954	inflammatory response	379	6	5.78	4.65301
GO:0071222	cellular response to lipopolysaccharide	113	4	12.92	4.8005
